# The efficacy and safety of probiotics intervention in preventing conversion of impaired glucose tolerance to diabetes: study protocol for a randomized, double-blinded, placebo controlled trial of the Probiotics Prevention Diabetes Programme (PPDP)

**DOI:** 10.1186/s12902-015-0071-9

**Published:** 2015-12-01

**Authors:** Qun Yan, Xu Li, Bo Feng

**Affiliations:** Department of Endocrinology, Shanghai East Hospital, Tongji University School of Medicine, Jimo Road 150, Shanghai, China

**Keywords:** Probiotics, Diabetes, Impaired glucose tolerance

## Abstract

**Background:**

Alterations in intestinal microbiota correlate with risk of development of obesity and type 2 diabetes. Probiotics have been suggested to play an important role in the management of dysglycemia, although the evidence is limited. In this study, we aim to explore the efficacy and safety of probiotics intervention in preventing type 2 diabetes in Chinese patients with impaired glucose tolerance.

**Methods/Design:**

A 24-month randomized intervention is conducted from January 2014 to December 2016. The target sample size for intervention is 200 middle-aged men and women aged 30–65 year-old with impaired glucose tolerance. Participants with persistent impaired glucose tolerance were assigned to group A (tablet A) and B (tablet B) in sequential order. The participants and investigators were blinded to the assignment. The primary outcome is development of diabetes. The secondary outcome measures include body composition, biochemical variables and the safety of the probiotics.

**Discussion:**

The results from this trial will provide the evidence on the efficacy and safety of probiotics administration in preventing conversion of impaired glucose tolerance to diabetes in a Chinese context.

**Trial registration:**

ChiCTRTRC13004024

## Background

Diabetes has become a worldwide epidemic in the general population. In China, the prevalence of diabetes among adults is 11.6 % according to the data released in 2013 [1]. In addition, the prevalence of prediabetes is up to 50.1 %. Individuals with prediabetes, especially impaired glucose tolerance (IGT), are at higher risk of developing type 2 diabetes, compared with individuals with normal glucose tolerance.

Studies have shown that early intervention in patients with IGT can significantly reduce the number of those patients who subsequently develop type 2 diabetes [2–6]. Those effective interventions conclude lifestyle modification and drug therapy. The efficacy of lifestyle modification in preventing the conversion of IGT to type 2 diabetes is proven [2, 3]. Drug therapy such as metformin, alpha-glucosidase inhibitor and Thiazolidinediones, has also been proven useful in the primary prevention of diabetes in people with IGT [4–9]. However, in clinical practice, the compliance of using hypoglycemic drugs to reduce the incidence of diabetes for nondaibetic subjects in natural population is relatively low. So to find a kind of non-hypoglycemic agents, on the basis of lifestyle modification, to help preventing the conversion of IGT to diabetes for subjects with prediabetes will be meaningful.

Recent evidences suggest that alterations in gut microbiota play an important role in the pathogenesis of obesity-related disorders, including diabetes and non-alcoholic fatty liver disease [10–15]. Compared with germ-free mice, conventionally raised mice were more prone to obesity and insulin resistance [10]. In human studies, it also has shown that gut microbiota composition differs between obese and lean subjects [11]. Recent metagenome-wide association studies showed that patients with type 2 diabetes have an increase in the abundance of Lactobacillus species and decreases in the abundance of Clostridium species [12, 13]. Thus, reshaping the gut microbiome may be a new direction for the prevention and treatment of diabetes in human beings. Currently the published evidences of the treatment of metabolic diseases with probiotics are limited, and the results are inconsistent [16–19]. Some but not all trials suggest that probiotics consumption may improve blood glucose level and antioxidant status in type 2 diabetic patients [16]. Two trials showed that probiotics intervention only improved the lipid metabolism but not blood glucose in patients with diabetes or gestational diabetes mellitus [17, 18]. Only one trial explored the effects of probiotics administration on the outcomes of blood lipids and blood pressure in patients with prediabetes, but no data on the outcome of glucose metabolism [19]. In addition, the intervention period of those trials may be too short (4 to 8 weeks) to observe the effect of probiotics. To date there is no clinical trial to explore whether probiotics administration can reduce the risk of development of type 2 diabetes in patients with prediabetes.

Therefore, the purpose of this study is to investigate the efficacy and safety of probiotics administration in preventing the development of type 2 diabetes in subjects with IGT compared to placebo. In addition, we will determine probiotics administration improve insulin sensitive and metabolic variables. The secondary outcome measures include body composition, biochemical variables and the safety of probiotics.

## Methods/Design

### Trial design

**Fig. 1:**
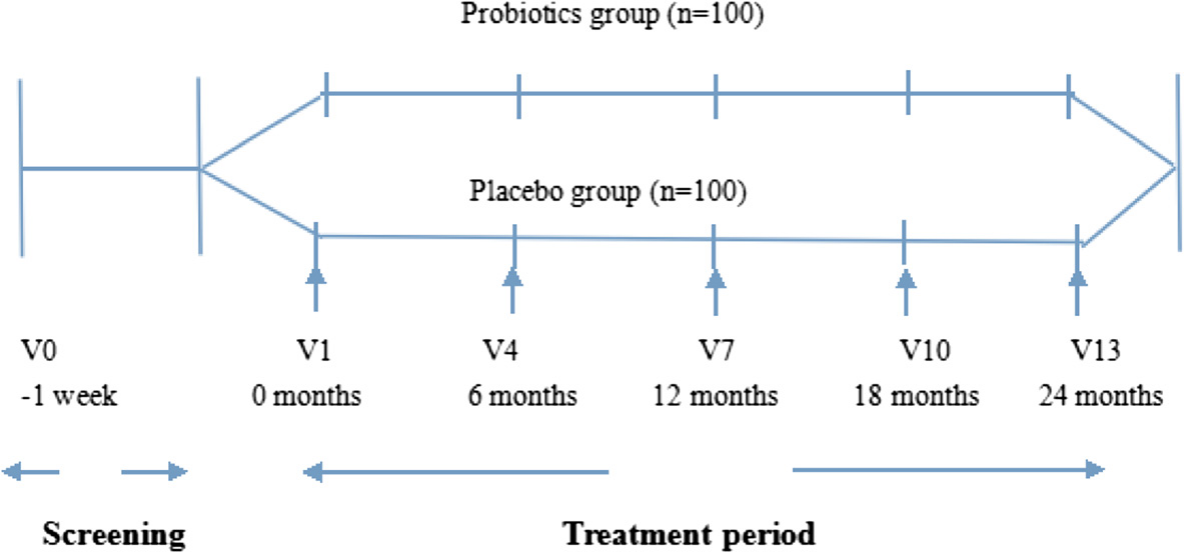
Study design and participant flow diagram for Probiotics Prevention Diabetes Programme (PPDP)

The PPDP is a 24-month, double-blinded, two-arm parallel group trial. Participants are individually randomized to one of two intervention groups in a 1:1 allocation. Participants are recruited from the outpatient department of five clinical centers in Shanghai during 2014 to 2016. They are Shanghai East Hospital, Shanghai Fifth People’s Hospital, Xinhua Hospital, Chongming Branch, Shanghai Pudong Hospital, Shanghai Seventh People’s Hospital. After an initial screening, potential subjects will be assessed for inclusion in the run-in trial. Those who meet the inclusion criteria are randomized to either intervention group or placebo group (Fig. 1).

### Participants

- ***Inclusion criteria are****:* (i) men or women aged 30–65 years; (ii) diagnose IGT after oral glucose tolerance test (OGTT) according to 1999 WHO criteria (fasting glucose <6.1 mmol/L, and 2-h post glucose load ≥ 7.8 and <11.1 mmol/L), (iii) no chronic cardiovascular, liver, kidney, lung diseases, (iv) informed consent, agreement on no other drug therapy on IGT during trial.
- ***Exclusion criteria are****:* (i) patients with severe liver, kidney, heart, nervous system and gastrointestinal diseases, (ii) diagnosis of diabetes mellitus, (iii) allergy to studied agents, (iv) suspected or definite a history of alcohol or drug abuse history, (v) the possibility of loss to follow-up according to the judgment of the researchers, (vi) patients who have participated in other clinical trials in the past 3 months.

### Interventions

Eligible participants are randomly assigned in double-blind to receive either probiotics or placebo which are blinded as Tablet A and Tablet B for 24 months. The key that indentifies the subjects and which group they belong to is kept by a third party, and is not revealed to the researchers until the end of the study and the statistic analysis is finished. Probiotics are provided by Bifico (Approval number: S10950032), an over the counter capsule consists of live combined Bifidobacterium longum [National Center for Medical Culture Collections (CMCC): P0001], Lactobacillus acidophilus [CMCC: P0002] and Enterococcus faecalis [CMCC: P0003] produced by Shanghai Sine Pharmaceutical Laboratories Co,Ltd. The study drug and the placebo are prepared in indistinguishable tablets. For both group, the dose are 840 mg daily, 210 mg per one pill, two pills per time, two times daily (Fig. 1).

### Outcomes

#### Primary outcomes

The primary clinical outcome is the improvement of risk of developing diabetes after administration of probiotics for 24 months.

#### Secondary outcomes

(i) the cumulative prevalence of diabetes in the two groups, (ii) the changes of blood glucose, lipid profile and insulin sensitivity in the two groups, (iii) the changes of body weight and waist circumference, (iv) the safety assessment of probiotics, (v) the change of the abundance of gut bacteria, (vi) the change of blood lipopolysaccharides (LPS) and GLP-1 levels.

### Trial procedures

#### Screening

The purpose of the screening is to verify the eligibility of potential participants selected from the Department of Outpatient. The recruitment of participants will be done in the Department of Endocrinology of Shanghai East Hospital, Shanghai, China. Every potential participant will be informed the detailed information of the objectives of the study, the participated risks and the expected benefits. When the concert form has been signed, participants will be asked to fill a standardized questionnaire about medical history and to undergo a 75 g OGTT, insulin release test, liver and kidney function test (blood draw after 8 h overnight fasting and 30 min and 2 h after glucose load).

#### First visit

Before the first visit, a physician examines all the results of the screening to verify whether the subject meets the inclusion criteria. In the first visit (within 1 week of the screening), if a person is qualified for participation in the study (fasting glucose <6.1 mmol/L, and 2-h post glucose load ≥7.8 and <11.1 mmol/L, with no other chronic diseases), he or she will be invited to the clinical laboratory of Department of Endocrinology, Shanghai East Hospital for height, weight, waist circumference and blood pressure measurement, as well as instructions for fecal sample. Then they will be randomized and assigned in double-blind to receive either probiotics or placebo for 8 weeks. We emphasize the importance of their participation in study and instruct them to take medicine on time during the intervention.

#### Follow-up visits

The purpose of follow-up visits is to monitor the compliance and to discover the adverse events. After the first visit, a researcher will contact participants every month to check their medication. The participants will have follow-up visits to the clinical laboratory every 2 months. Their height, weight, waist circumference, hip circumference, blood pressure, heart rate and newly combined medication will be checked at those time points. In addition, at months 6, 12, 18, 24, participants will undergo a 75 g OGTT to evaluate the glycemic status (blood draw after 8 h overnight fasting and 30 min and 2 h after glucose load) and take blood samples and fecal samples (Table 1).

**Table 1:** Visit study and efficacy variables for Probiotics Prevention Diabetes Programme (PPDP)

Visit number	1	2	3	4	5	6	7	8	9	10	11	12	13
Study month	0	2 m	4 m	6 m	8 m	10 m	12 m	14 m	16 m	18 m	20 m	22 m	24 m
Newly diagnosed diabetes(*n*)	×			×			×			×			×
OGTT + IRT(0 min,30 min,2 h)^a^	×			×			×			×			×
Total cholesterol	×			×			×			×			×
Triglyceride	×			×			×			×			×
High-density lipoprotein	×			×			×			×			×
Low-density lipoprotein	×			×			×			×			×
Body weight	×	×	×	×	×	×	×	×	×	×	×	×	×
Systolic blood pressure	×	×	×	×	×	×	×	×	×	×	×	×	×
Diastolic blood pressure	×	×	×	×	×	×	×	×	×	×	×	×	×
Waist circumference	×	×	×	×	×	×	×	×	×	×	×	×	×
Hip circumference	×			×			×			×			×
Abundance of bacteria	×			×			×			×			×
LPS	×			×			×			×			×
GLP-1	×			×			×			×			×

^a^OGTT, oral glucose tolerance test; IRT, insulin release test; LPS, lipopolysaccharides

### Methods of measurements

#### Physical examinations

Height and weight will be measured with light clothes and without shoes. Waist circumference will be measured at the level midway between the lower rib margin and the iliac crest. Hip circumference will be measured at the widest part of your buttocks. Body mass index (BMI) is calculated as weight (kg) divided by the square of height (m). Blood pressure will be measured twice after each subject had been seated for 10 min using a mercury electric sphygmomanometer. The average will be used for analysis.

#### Blood samples

OGTT will be performed after overnight fasting, 30 min and 2 h after the intake of 75 g glucose for the assessment of serum insulin and glucose levels. The blood samples were frozen at −80 °C until assayed. Venous blood samples will be collected after fasting over 8 h at 7 ~ 8 a.m. for the measurement of triglyceride and total cholesterol, high-density lipoprotein and low-density lipoprotein, and LPS and GLP-1 levels.

#### Stool samples collection

The stool samples will be collected at the first visit and at months 6, 12, 18, 24. Detailed stool sample collection and transportation instructions will be given by a study researcher. We will provide to subjects the materials for sample collection and transportation from home to hospital which including: 1 cool box, 1 sterile dressing room, 1 sterile EP tubes, 1 pair of sterile tweezer, 1 pair of plastic gloves and a plastic bag. The participants are advised to sample approximately 5 g of feces by tweezer attached to the sterile EP tubes. Then the sterile EP tubes are stored in a plastic bag in the cool box. After samples are collected, the subjects will immediately deliver the samples to the clinical laboratory in 1 h.

The stool samples will be frozen at −80 °C after collected and the DNA extraction will be performed using the E.Z.N.A.^®^ Stool DNA Kit (Omega Bio-tek, Norcross, GA, U.S.) according to manufacturer’s protocols. Fecal bacteria composition will be determined using 16S rRNA based methods and illumina MiSeq sequencing.

### Treatment compliance

The administration of study drugs will be recorded. Compliance will be discussed at each study visit and assessed based on returned tablet counts. Patients will be asked to return all unused study products including empty packages to the clinic at each visit.

### Sample size calculations

We will aim to recruit 100 subjects in each arm of the trial. Assuming a rate of 35 % developing diabetes in placebo group in China [20, 21], a type I error of 0.05 with two-sided, we have 80 % power to detect a 14 % difference in probiotics group at 2 year follow-up (accounting for our primary outcomes).

Considering a dropout rate of 10 %, the sample size for enrolled patients will rise up to 220 for all patients.

### Randomization

Participants are individually randomized to placebo or probiotics groups in a 1:1 allocation. Random number are generated by excel and overseen by an independent statistician. Research staff and participants remain blind to the allocated treatment group.

### Statistical methods

All analyses of end points are based on the intention-to-treat (ITT) principle. The results of the trial are reported as comparative summary statistics (difference in response rate or means) with 95 % confidence intervals. All statistical tests use a 5 % with two-sided significance level. Two-sided *t*-tests and chi-square tests are used to analyze the differences between the groups at baseline and during follow-up. For the primary outcome, survival curves are used to calculate the cumulative incidence of diabetes. The difference between the groups in the incidence of diabetes is tested by means of the two-sided log-rank test. For the secondary outcomes (the difference of the changes of blood glucose, lipid profile and insulin sensitivity, body weight, waist circumference, abundance of bacteria, levels of LPS and GLP-1 between the two groups), a mixed models are used on data collected at 6,12,18 and 24 months. An interaction between treatment group and time is fitted to allow estimation of treatment effects at each time point. The model adjusts for baseline variable and minimization factors. Linear Pearson’s correlation, partial correlation, and logistic regression analysis are used for analyzing relationships.

### Reporting of adverse events for this trial

We will report adverse events that might be reasonably related to probiotics. Adverse events are collected from time of signature of informed consent throughout the treatment period.

### Ethics, consent and permissions

The institutional review board of Shanghai East Hospital have reviewed and approved the protocol, ref: 2013–009. Trial procedures are compliant with the Declaration of Helsinki and the trial is registered with the Chinese Clinical Trials Register (ChiCTRTRC13004024). All subjects in this study are volunteers and complete a written informed form at the first visit. Each subject is given full and adequate oral and written information about the nature, purpose, possible risk and benefit of the study.

The present study will comply with the scientific ethics and integrity requirements in scientific and clinical practice.

### Consent to publish

We have obtained consent to publish from the participant to report individual patient data.

## Discussion

IGT is associated with progressing to diabetes and risk of cardiovascular diseases. Epidemiological studies showed that 25 % of patients with IGT progress to type 2 diabetes in 5 years [2] and the rate reach to 41 % in China [20]. An unhealthy diet and the alterations in gut microbiota play an important role in this process. Although the diet control is helpful to the prevention and treatment of diabetes, it is difficult for the individual to insist on. Recent studies showed that reshaping the gut microbiome can improve the energy metabolism [16–19, 22], which provide a new insight on preventing the occurrence of type 2 diabetes. Therefore, we present a design of a randomized double-blinded, placebo-controlled trial to investigate the efficacy and safety of probiotics intervention in preventing conversion of IGT to type 2 diabetes in China. To the best of our knowledge, this is the first study, in a relative larger sample and in a longer period, to evaluate the efficacy of probiotics administration on glucose metabolism in IGT subjects.

This trial has been designed to address the limitations of previous studies of the effects of probiotics administration on the outcomes of metabolic diseases. The duration of intervention and the sample size will allow us to explore the effects of probiotics intervention on metabolic diseases. We expect that the overall incidence of diabetes will be significantly reduced in probiotics group than placebo group, which suggest that type 2 diabetes can be prevented by oral administration probiotics for subjects at high risk for the disease. We also anticipate that probiotics intervention will improve insulin sensitivity, lipid profile, and inflammatory state by changing the gut microbiota composition.

This trial using oral administration of probiotics offers a new approach for both diabetes and metabolic diseases prevention. If the result of the research shown to be effective, it will open a new strategy for diabetes prevention.

### Trial status

Participant recruitment started in September 2014. Baseline measurement started in October 2014 for the first enrolled subjects. Medicine interventions started in October 2014. The recruitment of subjects is continuing until October 2015.

## Abbreviations

PPDP: Probiotics prevention diabetes programme
IGT: Impaired glucose tolerance
OGTT: Oral glucose tolerance test
CMCC: National center for medical culture collections
LPS: Lipopolysaccharides
BMI: Body mass index
ITT: Intention-to-treat
